# A simple protocol for isolating microglia from adult mouse brain

**DOI:** 10.1515/nipt-2023-0014

**Published:** 2023-08-02

**Authors:** Sudipta Chakrabarti, Sukhamoy Gorai, Kalipada Pahan

**Affiliations:** Department of Neurological Sciences, Rush University Medical Center, Chicago, USA

**Keywords:** activation, adult mouse brain, microglia, neurodegeneration, phagocytosis

## Abstract

**Objectives:**

Although microglia are activated in adult and aged brains resulting in neurodegenerative and neuroinflammatory disorders, most of the cell culture studies on microglia deal with neonatal microglia because of ease of isolation. Microglia could be isolated from adult brains, but it requires separation by density gradient centrifugation, magnetic beads, etc. Here, we describe a simple protocol of isolating highly purified microglia from adult mouse brains.

**Methods:**

Our protocol involves dilution with sterile PBS or media, regular centrifugation, and plating on poly-D-lysine-coated flasks.

**Results:**

These adult microglia expressed the inducible nitric oxide synthase in response to preformed α-syn fibril, an etiological reagent of Parkinson’s disease, and bacterial lipopolysaccharides, one of the prototype proinflammatory stimuli. Moreover, these adult microglia exhibited phagocytosis, which was stimulated by LPS treatment.

**Conclusions:**

These results suggest that adult microglia isolated by our procedure are functional and that these adult microglia could be used for studies related to neurodegenerative disorders.

## Introduction

Microglia, the innate immune cells of the central nervous system (CNS), are considered as key respondents of CNS defense networks like phagocytes [1, 2]. While in one hand, microglia destroy pathogens and clear unwanted materials from the brain [3], upon an insult or injury, these cells also secrete wide range of proinflammatory molecules including cytokines, chemokines, reactive oxygen species, nitric oxide, etc. [3–5]. These soluble proinflammatory mediators from microglia are also capable of activating astrocytes and causing damage to neurons and myelin-producing cells oligodendrocytes [6, 7]. Therefore, activation of microglia are nowadays considered as a hallmark of different neurodegenerative and neuroinflammatory disorders including Alzheimer’s disease, Parkinson’s disease, Huntington’s disease, amyotrophic lateral sclerosis, multiple sclerosis, traumatic brain injury, etc. [8–13]. Since these disorders mainly affect adult and aged population, it can be said that microglia are activated mainly in adult brains.

On the other hand, most of the studies on microglia are being carried out in either microglial cell line or primary microglia isolated from mouse pups [14–20]. Although there are some established protocols for the isolation of microglia from adult mouse brains, these are based on magnetic bead, flow cytometry, density gradient centrifugation, or other tedious techniques [21–24]. Here we describe a simple and reproducible method for isolating highly purified microglia from adult mouse brain tissue. In the first part of this study, we optimized the isolation procedure and characterized the isolated adult mouse primary microglia based on various markers. In the second part of the study, we exhibited the utility of isolated adult microglia for studying microglial activation/inflammation followed by stimulation with preformed α-syn fibril (α-syn PFF) and bacterial lipopolysaccharides (LPS). These adult microglia also exhibited phagocytosis. The availability of functional and highly pure microglia from adult brains may be helpful for developing therapies for different neurodegenerative disorders in which microglial activation plays a pivotal role in disease pathogenesis.

## Materials and methods

### Reagents

Phosphate buffer saline (PBS), trypsin, DMEM/F-12, and antibodies against CD68 were purchased from Thermo Fisher Scientific (Waltham, MA). Fetal bovine serum (FBS) was bought from Atlas Biologicals (Fort Collins, CO). Antibiotic-antimycotic was purchased from Sigma (St. Louis, MO). Recombinant human α-syn was purchased from Anaspec (Fremont, CA). Antibodies against anti-goat Iba1 were purchased from Abcam (Waltham, MA). Dako antibodies against glial fibrillary acidic protein (GFAP) were bought from Agilent (Santa Clara, CA). Antibodies against myelin basic protein (MBP) and micro-tubule associated protein 2 (MAP2) were purchased from Santa Cruz Biotechnology (Santa Cruz, CA, USA). Cy2- and Cy5-conjugated antibodies were obtained from Jackson Immuno-Research Laboratories (West Grove, PA).

### Isolation of adult mouse primary microglia

**Figure 1:**
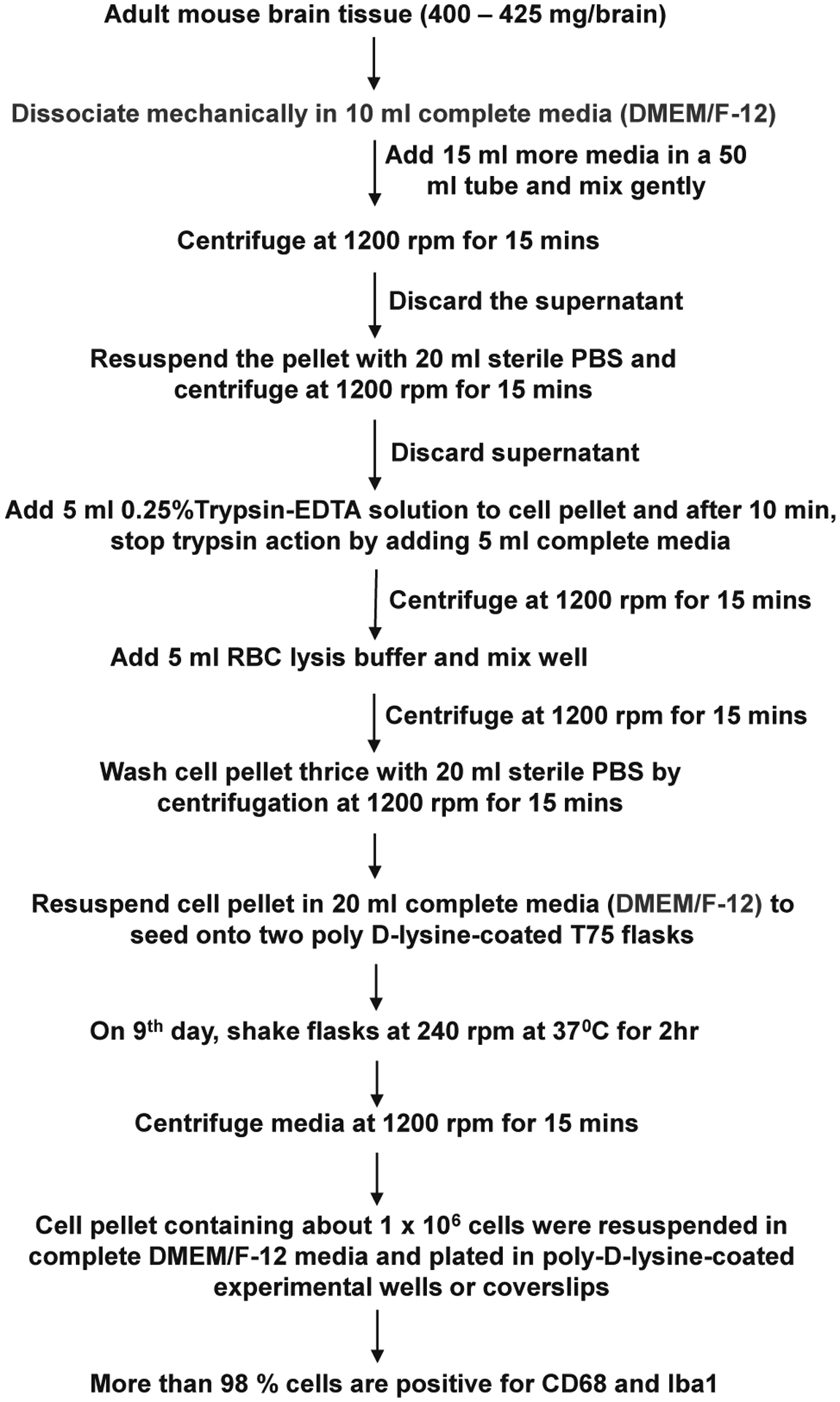
Detailed flowchart for the isolation of microglia from adult mice.

C57/BL6J mice were purchased from Jackson Laboratories (Bar Harbor, ME). All the experimental protocols were reviewed and approved by the Institutional Review Board of the Rush University Medical Center. Adult C57/BL6 J mice (5–6 months old) of both sexes were used for the isolation of microglia as described by us and others for neonatal microglia [14, 20, 25–27] with modifications. Mice were anesthetized with ketamine/xylazine injectable followed by decapitation. One adult mouse brain (weight 400–425 mg) was meshed by a pestle in a cell strainer with 10 mL of complete DMEM/F-12 media and transferred to a 50 mL falcon tube followed by dilution with another 15 mL of complete DMEM/F-12 media (Figure 1). Cell suspension was centrifuged at 1200 rpm for 15 min. Pellet was resuspended in 20 mL sterile PBS followed by centrifugation. Cell pellet was digested with 5 mL 0.25 % trypsin-EDTA solution for 10 min followed by addition of 5 mL complete media for stopping the activity of trypsin. After centrifugation, 5 mL RBC lysis buffer (Sigma, St. Louis, MO) was added to the pellet to remove red blood cells. Cell suspension was centrifuged followed by washing of cell pellet thrice with sterile PBS. Then cell pellet was suspended in complete media and seeded onto poly-D-lysine-coated flasks. Media was changed every three day and on ninth day, flasks were shaken at 240 rpm at 37 °C for 2 h to get microglia. After shaking, media was centrifuged and cell pellet was resuspended in complete DMEM/F-12 media to plate on poly-D-lysine-coated wells or coverslips for experiments.

### Preparation of α-syn fibrils

Human α-syn fibrils were prepared as described before [26, 28]. Briefly, human α-syn monomers solubilized in 30 mM Tris-HCl (pH 7.4) at 350 μM concentration were rotated continuously in a rotary shaker at 250 rpm at 37 °C for 7 days. Formation of α-syn fibril was validated by transmission electron microscopy [26, 28] and Western blotting [26] with anti-α-syn antibodies (BD Bioscience, San Jose, CA).

### Treatment of adult microglia with LPS and preformed α-syn fibrils (PFF)

Based on previous studies [10, 26, 29], microglia were treated with 1 μg/mL LPS and 25 nM PFF, separately, in serum-free DMEM/F-12 media.

### Immunostaining

Immunostaining was performed as described earlier [30, 31]. Briefly, coverslips containing 200–300 cells/mm^2^ were fixed with 4 % paraformaldehyde for 15 min, followed by treatment with cold methanol (−20 °C) for 5 min and two rinses in PBS. Samples were blocked with 2 % BSA in PBS containing Tween 20 (PBST) for 30 min followed by incubation in PBST containing 1 % BSA and mouse anti-CD68 (1:200), goat anti-IBA1 (1:200), rabbit anti-iNOS (1:200), mouse anti-GFAP (1:500), mouse anti-MBP (1:200), and goat anti-MAP-2 (1:100). After three washes in PBST (15 min each), slides were further incubated with Cy2, Cy5, and DAPI (Jackson ImmunoResearch, West Grove, PA, USA). After secondary antibody incubation, coverslips were rinsed in 1X PBS, mounted on slides in Fluoromount *(Sigma)* and imaged using an *Olympus BX41* fluorescent microscope equipped with a *Hamamatsu ORCA-03G* camera.

### Phagocytosis assay

Primary microglia isolated from WT adult mice were plated on poly-D-lysine-coated glass coverslips. Cells were treated with latex beads-rabbit IgG-FITC complex (Cayman Chemical, Ann Arbor, MI) at a dilution of 1:200 for 30 min. For LPS-stimulated microglia, cells were stimulated with 1 μg/mL LPS for 1 h under serum-free condition, followed by adding IgG-FITC complex and monitoring phagocytosis by immunostaining [14, 26]. Briefly, cells were washed at least three times with a warm (37 °C) serum-free medium and then fixed with 4 % paraformaldehyde. Fixed cells were processed for Iba1 immunostaining using the procedure described above. Imaging was performed under Olympus BX41 fluorescence microscope.

### Statistical analysis

Data are expressed as mean ± SD. Statistical comparisons were made using Student’s t test (The SAS system, Caly, NC, USA). Differences between means were considered significant when p<0.05.

## Results

### Isolation of microglia from adult mouse brains

**Figure 2:**
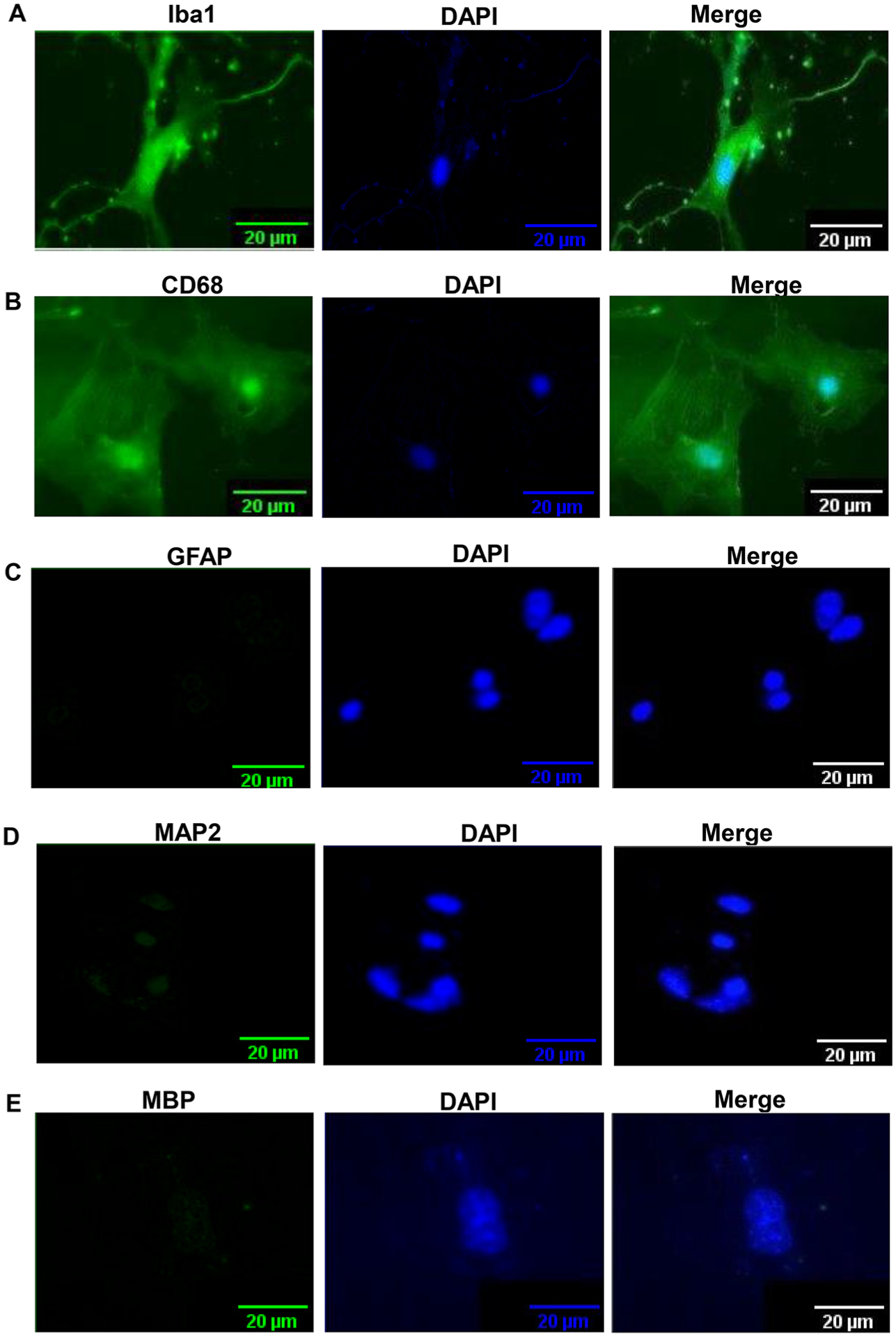
Purity of microglia isolated from adult mouse brain. Cells were immunostained with antibodies against Iba-1 (A), CD68 (B), GFAP (C), MAP2 (D), and MBP (E). DAPI was used for visualizing nuclei. Results represent three independent experiments.

Our detailed procedure for the isolation of highly purified microglia from 5–6-month-old mice is summarized in Figure 1. Since adult brains are very rich in different complex lipids, we employed dilution with DMEM/F-12 media or sterile PBS to centrifuge down mixed glial cells (Figure 1). While for the centrifugation of neonatal brain cells, we regularly use 1000 rpm for 5 min [14, 32], for adult brain cells, we increased the duration at a little higher rotational speed (1200 rpm for 15 min) (Figure 1). Moreover, in contrast to neonatal brain cells, adult brain cells have a tendency to poorly adhere to the surface of tissue culture wells and plates. Therefore, we used poly-D-lysine-coated flasks for better adherence of adult mixed glial cells. Immunostaining results show that most of the cells isolated by this procedure (Figure 1) were positive for either Iba1(Figure 2A) or CD68 (Figure 2B). On the other hand, by immunostaining with antibodies against GFAP (Figure 2C), MAP2 (Figure 2D) and MBP (Figure 2E), we could not detect any astrocyte, neuron and oligodendrocyte in primary adult microglia, indicating the isolation of highly pure microglia. From each adult mouse brain, approximately 1 million microglia were obtained (Figure 1).

### Induction of iNOS in primary adult microglia

**Figure 3:**
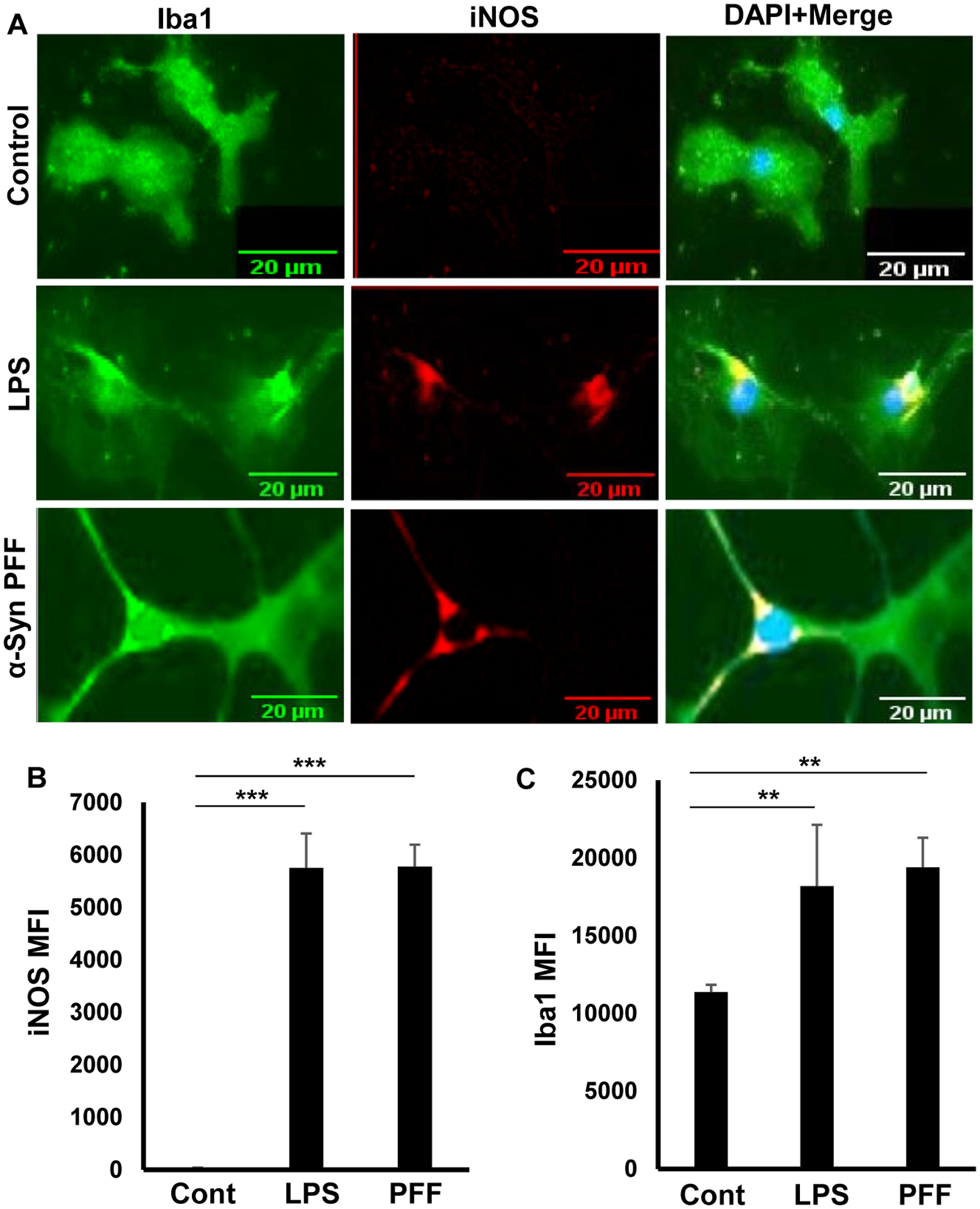
Induction of iNOS in adult mouse microglia by bacterial LPS and α-syn PFF. Microglia were stimulated with either LPS (1 μg/mL) or α-syn PFF (25 nM), (A) under serum-free condition for 24 h followed by double-label immunofluorescence with antibodies against iNOS (Red) and IBA1 (green). DAPI was used to visualize nuclei. Mean fluorescence intensity (MFI) of iNOS, (B) and Iba1, (C) was calculated from three different experiments (3 images per experiment). Results were statistically analyzed by Student’s t test. **p<0.01 & ***p<0.001.

Nitric oxide produced by inducible nitric oxide synthase (iNOS) in adult microglia in response to various proinflammatory stimuli plays an important role in the pathophysiology of neurodegenerative diseases like Alzheimer’s disease, Parkinson’s disease and multiple sclerosis [5, 33–37]. While bacterial lipopolysaccharides (LPS) has been being used as one of the model proinflammatory stimuli for decades [29, 32], preformed α-syn fibril (PFF) is now considered as an etiological reagent of PD [38]. Therefore, to examine whether adult microglia were functionally active, these cells were stimulated by LPS and PFF, separately, followed by double-labeling with antibodies against Iba1 and iNOS. Although unstimulated adult microglia did not express any iNOS, upon challenge with both LPS and PFF, marked expression of iNOS was observed in primary adult microglia (Figure 3A). These results were corroborated by quantification of mean fluorescence intensity (MFI) of iNOS (Figure 3B). Moreover, as expected, stimulation with both LPS and PFF led to increase in Iba1 (Figure 3C). These results suggest that adult microglia isolated by our procedure could be activated by proinflammatory stimuli.

### Phagocytosis in primary adult microglia

**Figure 4:**
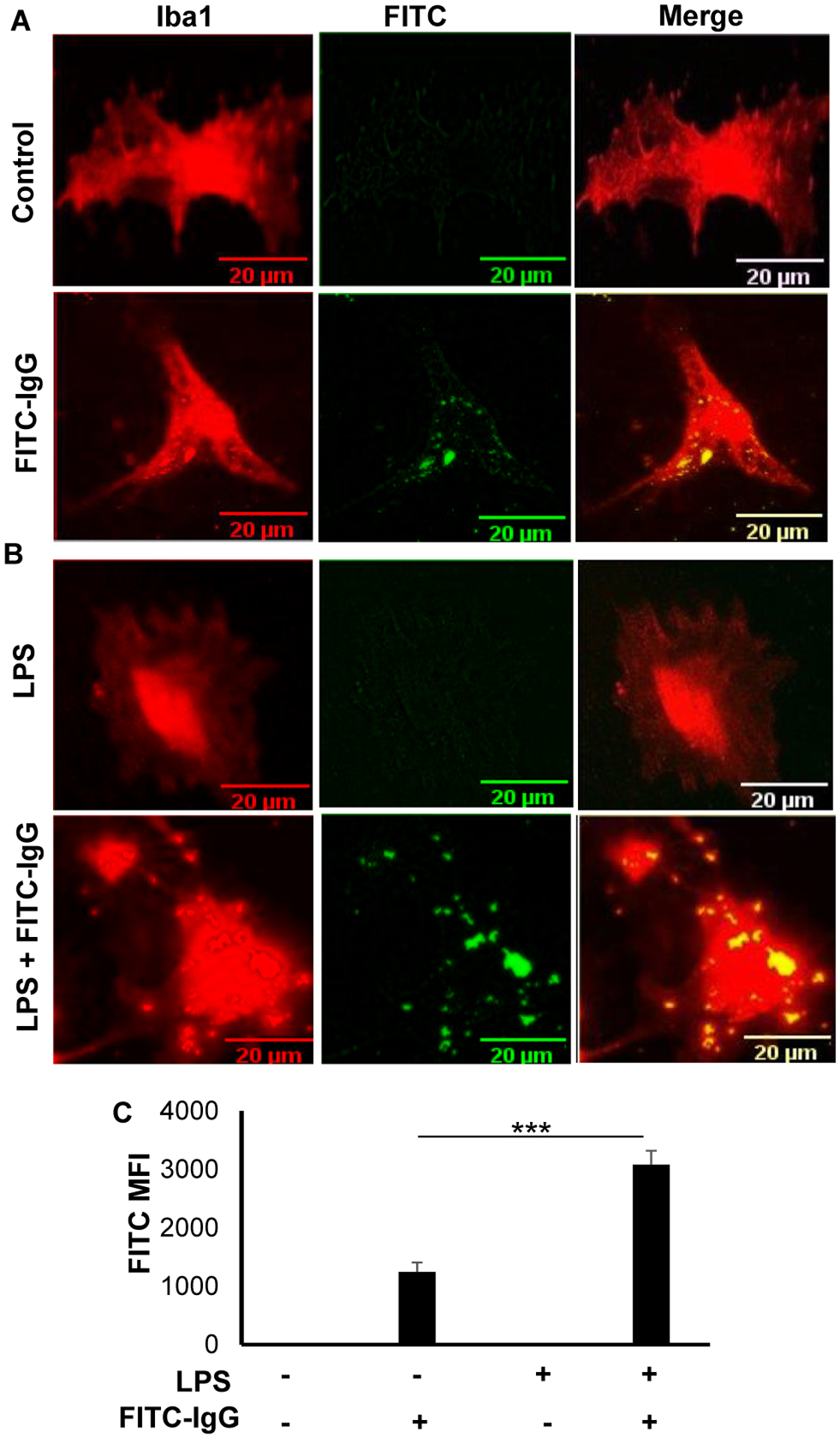
Phagocytic activity of adult mouse microglia. (A) Microglia were treated with latex beads-rabbit IgG-FITC complex at a dilution of 1:200 for 30 min under serum-free condition followed by immunolabeling with antibodies against Iba1. (B) Microglia were treated with 1 μg/mL LPS for 1 h under serum-free condition followed by addition of latex beads-rabbit IgG-FITC complex. After 30 min, cells were immunostained with antibodies against Iba1. (C) MFI of FITC was calculated from three different experiments (3 images per experiment). Results were statistically analyzed by Student’s t test. ***p<0.001.

Microglia are known to exhibit a spectrum of phenotypes ranging from ameboid to ramified depending on the challenges [39–41]. While ramified microglia have many processes that facilitate the interaction with nearby neurons, astrocytes and blood vessels [42], amoeboid microglia have retracted processes and greatest capacity for phagocytosis [43]. We examined whether adult microglia isolated by our procedure retained the phagocytic property as monitored by using fluorescein labelled IgG-tagged latex beads. Unstimulated microglia exhibited some phagocytic property as evident from the engulfment of FITC-IgG-latex beads (Figure 4A & C). However, significant (p<0.001) increase in phagocytosis was observed in adult microglia upon LPS stimulation (Figure 4B & C). These results suggest that adult microglia could be used phagocytosis-related studies.

## Discussion

It is important to describe experimental condition mimicking the diseased condition at a cellular level. Since most of the neurodegenerative disorders involve microglial activation in adult brains, for better mimicking the *in vivo* situation in neurodegenerative brains, it is important to isolate microglia from adult brains. However, cell culture studies involving microglia mostly use neonatal microglia due to ease of isolation. Here, we delineate a simple and easy procedure for the isolation of very pure (>98 %) microglia from adult brains. We used poly-D-lysine-coated flasks for plating adult mixed glial cells and probably due to this reason, we have not seen any astrocytes with microglia after shaking on ninth day.

There are some published protocols for the isolation of adult microglia. For example, Lee and Tansey [23] used enzymatic dissociation with dispase II, papain and DNase I followed by mechanical dissociation and Percoll gradient centrifugation of various densities. Percoll gradient centrifugation and anti-myelin magnetic beads were used by Nikodemova and Watters [24] for the isolation and purification of adult microglia. Grabbert and McColl [44] employed Percoll density gradient centrifugation and immunomagnetic separation using anti-CD11b microbeads to isolate adult microglia. On the other hand, Pan and Wang [45] used magnetic activated cell sorting) and fluorescence activated cell sorting procedures to isolate adult microglia. However, our protocol does not involve any density gradient centrifugation and immunomagnetic bead separation. To separate brain cells from myelin/lipids, we employed dilution to reduce the density of lipids followed by centrifugation. Since it is difficult to spin down adult brain cells, we used 1200 rpm for 15 min for centrifugation in all the steps. Moreover, in contrast to neonatal brain cells, adult brain cells have a tendency to adhere to cell culture plates loosely. To overcome this issue, we used poly-D-lysine-coated flasks for plating adult mixed glial cells and probably due to this reason, we have not seen any astrocytes with microglia after shaking on ninth day. Astrocytes were strongly attached to poly-D-lysine-coated surfaces, which were removed only by trypsinization (for other study).

Microglia are important mediators of immune modulated neuronal cell death in the CNS [46, 47]. Mounting evidence suggests that the neuroimmune modulation by microglia contributes to the pathogenesis of several neurodegenerative disorders like AD, PD, HD, etc. The triggering events that precede microglial inflammation is under intense study. Microglial inflammation can be triggered by numerous endogenous and exogenous sources including proinflammatory molecules, pesticides, insoluble protein aggregates, and even chemical signals from damaged neurons themselves. Upon inflammatory stimulation, activated microglia produce various cytotoxic factors such as superoxide, nitric oxide, TNFα, IL-6, IL-1β, etc. to potentiate an insidious cycle of inflammation resulting in progressive neuronal injury in neurodegenerative disorders. It is important to see that adult mouse microglia isolated by dilution and centrifugation responded to LPS and α-syn PFF to express iNOS. In addition to the induction of proinflammatory molecules, microglia are the only cells in the CNS that are well-equipped for phagocytosis [48]. It has been shown that microglial phagocytosis is downregulated in neurodegenerative disorders like AD, PD, etc. [49]. Interestingly, the phagocytic property is present in adult mouse microglia isolated by our procedure that was also stimulated by LPS challenge. Therefore, these adult microglia may be used for studies related to microglial activation/inflammation as well as phagocytosis.

In brief, we have provided a simple method of isolation and cultivation of highly purified microglia from the adult mouse brain tissue. These microglia express their characteristic cell surface markers and carry out their prototype functions as they do in physiological and pathophysiological situations. Therefore, our protocol of isolating adult microglia may be helpful for researchers for studying the biology and medicine of adult microglia.
